# Draft Genome Sequence of an Anaerobic Ammonium-Oxidizing Bacterium, “*Candidatus* Brocadia sinica”

**DOI:** 10.1128/genomeA.00267-15

**Published:** 2015-04-16

**Authors:** Mamoru Oshiki, Kaori Shinyako-Hata, Hisashi Satoh, Satoshi Okabe

**Affiliations:** a Department of Civil Engineering, Nagaoka National College of Technology, Nagaoka, Niigata, Japan; b Faculty of Engineering, Division of Environmental Engineering, Hokkaido University, Sapporo, Hokkaido, Japan

## Abstract

A draft genome sequence of an anaerobic ammonium-oxidizing (anammox) bacterium, “*Candidatus* Brocadia sinica,” was determined by pyrosequencing and by screening a fosmid library. A 4.07-Mb genome sequence comprising 3 contigs was assembled, in which 3,912 gene-coding regions, 47 tRNAs, and a single *rrn* operon were annotated.

## GENOME ANNOUNCEMENT

Anaerobic ammonium oxidation (anammox) is a microbial process in which NH_4_^+^ and NO_2_^−^ are converted to N_2_ gas via N_2_H_4_ (1). The anammox process is mediated by monophyletic bacteria deeply branched in the phylum *Planctomycetes* (2). Anammox bacteria are affiliated with the order *Brocadiales*, which contains five candidate genera: “*Candidatus* Kuenenia,” “*Candidatus* Brocadia,” “*Candidatus* Anammoxoglobus,” “*Candidatus* Jettenia,” and “*Candidatus* Scalindua” (3). Draft genome sequences have been determined from anammox bacteria affiliated with “*Ca.* Kuenenia” (4, 5), “*Ca.* Jettenia” (6, 7), and “*Ca.* Scalindua” (8, 9). As for “*Ca.* Brocadia,” the draft genome sequence of “*Ca.* Brocadia fulgida” was previously determined (10); the genome comprised 2,786 contigs, in which large parts of the genes were fragmented.

We had initially enriched an anammox bacterium affiliated with “*Ca.* Brocadia,” namely, “*Ca*. Brocadia sinica,” from activated sludge by using a continuous up-flow column reactor (11). “*Ca.* Brocadia sinica” cells were further enriched in a membrane bioreactor (>94% of the total biomass as determined by fluorescence *in situ* hybridization analysis) (12). This anammox bacterium shares only 94% 16S rRNA gene sequence similarity with “*Ca.* Brocadia fulgida” and has a relatively high growth rate (i.e., *μ*_max_ = 0.0041 h^−1^) (13). The objective of this study was to determine a high-quality “*Ca.* Brocadia sinica” genome by metagenomic sequencing and to provide a genetic platform for this anammox bacterium.

Genomic DNA of “*Ca.* Brocadia sinica” was extracted using a Genomic-tip 500/G kit (Qiagen KK, Japan, Tokyo Japan) and subjected to single and 3-kb paired-end pyrosequencing using a GS FLX titanium sequencer (Roche Diagnostics KK, Tokyo, Japan). The single and 3-kb paired-end pyrosequencing produced 628,274 (total 215,259,727 bp) and 576,054 reads (189,603,440 bp), respectively, which were assembled into 18 scaffolds using GS *De Novo* Assembler version 2.6 software (Roche Diagnostics KK, Tokyo, Japan). A 35-kb insert fosmid library was constructed using the genomic DNA and pCC1FOS vector (Illumina, Madison, WI, USA), and 1,632 clones were screened by PCR amplification. The PCR products covering the regions of 15 missing links were obtained, and the gaps in the scaffolds were filled by standard primer-walking and Sanger sequencing techniques (14). A 4.07-Mb genome sequence comprising 3 contigs (42.41% G+C content) was obtained, and 3,912 gene-coding regions, 47 tRNAs, and a single *rrn* operon were annotated. Completeness was estimated to be 98.3% based on the presence/absence of 180 core genes (15).

The “*Ca.* Brocadia sinica” genome encodes gene clusters for core anammox metabolism, including 10 and 2 copies of *hao-hdh* and *hzs* encoding hyrdoxylamine dehydrogenease/hydrazine dehydrogenase and hydrazine synthase, respectively. Conversely, *nirS* and *nirK* encoding cytochrome *cd_1_*-type or copper-containing NO-forming nitrite reductase were missing in the “*Ca.* Brocadia sinica” genome as well as the “*Ca.* Brocadia fulgida” genome (10), suggesting that “*Ca.* Brocadia” cells employ an unidentified nitrite reductase for NO_2_^−^ reduction in the anammox process, which should be further investigated.

### Nucleotide sequence accession numbers. 

The “*Ca.* Brocadia sinica” genome was deposited in the DDBJ nucleic acid sequence database under the accession numbers BAFN01000001 through BAFN01000003.

## References

[1] Kartal B, de Almeida NM, Maalcke WJ, Op den Camp HJ, Jetten MS, Keltjens JT 2013 How to make a living from anaerobic ammonium oxidation. FEMS Microbiol Rev 37:428–461. doi:10.1111/1574-6976.12014. 23210799

[2] Strous M, Fuerst JA, Kramer EH, Logemann S, Muyzer G, van de Pas-Schoonen KT, Webb R, Kuenen JG, Jetten MS 1999 Missing lithotroph identified as new planctomycete. Nature 400:446–449. doi:10.1038/22749. 10440372

[3] Jetten MSM, op den Camp HJM, Kuenen JG, Strous M 2009 Family I. “*Candidatus* Brocadiaceae” fam. nov., p 918–925. *In* Krieg NR, Staley JT, Brown DR, Hedlund BP, Paster BJ, Ward NL, Ludwig W, Whitman WB (ed), Bergey’s manual of systematic bacteriology: the *Bacteroidetes*, *Spirochaetes*, *Tenericutes* (*Mollicutes*), *Acidobacteria*, *Fibrobacteres*, *Fusobacteria*, *Dictyoglomi*, *Gemmatimonadetes*, *Lentisphaerae*, *Verrucomicrobia*, *Chlamydiae*, and *Planctomycetes*, vol 4, 2nd ed. Springer-Verlag, New York, NY.

[4] Strous M, Pelletier E, Mangenot S, Rattei T, Lehner A, Taylor MW, Horn M, Daims H, Bartol-Mavel D, Wincker P, Barbe V, Fonknechten N, Vallenet D, Segurens B, Schenowitz-Truong C, Médigue C, Collingro A, Snel B, Dutilh BE, op den Camp HJ, van der Drift C, Cirpus I, van de Pas-Schoonen K, Harhangi H, van Niftrik L, Schmid M, Keltjens J, van de Vossenberg J, Kartal B, Meier H, Frishman D, Huynen M, Mewes H, Weissenbach J, Jetten M, Wagner M, Le Paslier D 2006 Deciphering the evolution and metabolism of an anammox bacterium from a community genome. Nature 440:790–794. doi:10.1038/nature04647. 16598256

[5] Speth DR, Hu B, Bosch N, Keltjens JT, Stunnenberg HG, Jetten MS 2012 Comparative genomics of two independently enriched “*Candidatus* Kuenenia stuttgartiensis” anammox bacteria. Front Microbiol 3:307. doi:10.3389/fmicb.2012.00307. 22934093 PMC3423927

[6] Hira D, Toh H, Migita CT, Okubo H, Nishiyama T, Hattori M, Furukawa K, Fujii T 2012 Anammox organism KSU-1 expresses a NirK-type copper-containing nitrite reductase instead of a NirS-type with cytochrome *cd*_1_. FEBS Lett 586:1658–1663. doi:10.1016/j.febslet.2012.04.041. 22673575

[7] Hu Z, Speth DR, Francoijs KJ, Quan ZX, Jetten MS 2012 Metagenome analysis of a complex community reveals the metabolic blueprint of anammox bacterium “*Candidatus* Jettenia asiatica.” Front Microbiol 3:366. doi:10.3389/fmicb.2012.00366. 23112795 PMC3482989

[8] Van de Vossenberg J, Woebken D, Maalcke WJ, Wessels HJ, Dutilh BE, Kartal B, Janssen-Megens EM, Roeselers G, Yan J, Speth D, Gloerich J, Geerts W, van der Biezen E, Pluk W, Francoijs K-J, Russ L, Lam P, Malfatti SA, Tringe SG, Haaijer SC, op den Camp HJM, Stunnenberg HG, Amann R, Kuypers MMM, Jetten MSM 2013 The metagenome of the marine anammox bacterium “*Candidatus* Scalindua profunda” illustrates the versatility of this globally important nitrogen cycle bacterium. Environ Microbiol 15:1275–1289. doi:10.1111/j.1462-2920.2012.02774.x. 22568606 PMC3655542

[9] Speth DR, Russ L, Kartal B, op den Camp HJ, Dutilh BE, Jetten MS 2015 Draft genome sequence of anammox bacterium “*Candidatus* Scalindua brodae,” obtained using differential coverage binning of sequencing data from two reactor enrichments. Genome Announc 3(1):e01415-14. doi:10.1128/genomeA.01415-14. 25573945 PMC4290996

[10] Gori F, Tringe SG, Kartal B, Machiori E, Jetten MSM 2011 The metagenomic basis of anammox metabolism in *Candidatus* “Brocadia fulgida.” Biochem Soc Trans 39:1799–1804. doi:10.1042/BST20110707. 22103529

[11] Tsushima I, Ogasawara Y, Kindaichi T, Satoh H, Okabe S 2007 Development of high-rate anaerobic ammonium-oxidizing (anammox) biofilm reactors. Water Res 41:1623–1634. doi:10.1016/j.watres.2007.01.050. 17350073

[12] Oshiki M, Awata T, Kindaichi T, Satoh H, Okabe S 2013 Cultivation of planktonic anaerobic ammonium oxidation (anammox) bacteria using membrane bioreactor. Microbes Environ 28:436–443. doi:10.1264/jsme2.ME13077. 24200833 PMC4070702

[13] Oshiki M, Shimokawa M, Fujii N, Satoh H, Okabe S 2011 Physiological characteristics of the anaerobic ammonium-oxidizing bacterium “*Candidatus* Brocadia sinica.” Microbiology 157:1706–1713. doi:10.1099/mic.0.048595-0. 21474538

[14] Sambrook J, Russell DW 2001 Molecular cloning: a laboratory manual, 3rd ed. Cold Spring Harbor Laboratory Press, Cold Spring Harbor, NY.

[15] Martín HG, Ivanova N, Kunin V, Warnecke F, Barry KW, McHardy AC, Yeates C, He S, Salamov AA, Szeto E, Dalin E, Putnam NH, Shapiro HJ, Pangilinan JL, Rigoutsos I, Kyrpides NC, Blackall LL, McMahon KD, Hugenholtz P 2006 Metagenomic analysis of two enhanced biological phosphorus removal (EBPR) sludge communities. Nat Biotechnol 24:1263–1269. doi:10.1038/nbt1247. 16998472

